# Assessing implementation fidelity in the First Episode Rapid Early Intervention for Eating Disorders service model

**DOI:** 10.1192/bjo.2021.51

**Published:** 2021-05-07

**Authors:** Katie L. Richards, Michaela Flynn, Amelia Austin, Katie Lang, Karina L. Allen, Ranjeet Bassi, Gabrielle Brady, Amy Brown, Frances Connan, Mary Franklin-Smith, Danielle Glennon, Nina Grant, William Rhys Jones, Kuda Kali, Antonia Koskina, Kate Mahony, Victoria A. Mountford, Nicole Nunes, Monique Schelhase, Lucy Serpell, Ulrike Schmidt

**Affiliations:** Department of Psychological Medicine, Institute of Psychiatry, Psychology and Neuroscience, King's College London, UK; Department of Psychological Medicine, Institute of Psychiatry, Psychology and Neuroscience, King's College London, UK; Department of Psychological Medicine, Institute of Psychiatry, Psychology and Neuroscience, King's College London, UK; Department of Psychological Medicine, Institute of Psychiatry, Psychology and Neuroscience, King's College London, UK; Department of Psychological Medicine, Institute of Psychiatry, Psychology and Neuroscience, King's College London, UK; and Eating Disorder Outpatient Service, South London and Maudsley NHS Foundation Trust, UK; Eating Disorder Outpatient Service, South London and Maudsley NHS Foundation Trust, UK; Vincent Square Eating Disorder Service, Central and North West London NHS Foundation Trust, UK; Eating Disorder Outpatient Service, South London and Maudsley NHS Foundation Trust, UK; Vincent Square Eating Disorder Service, Central and North West London NHS Foundation Trust, UK; Eating Disorder Service, Leeds and York Partnership NHS Foundation Trust, UK; Eating Disorder Outpatient Service, South London and Maudsley NHS Foundation Trust, UK; Eating Disorder Outpatient Service, South London and Maudsley NHS Foundation Trust, UK; Eating Disorder Service, Leeds and York Partnership NHS Foundation Trust, UK; Vincent Square Eating Disorder Service, Central and North West London NHS Foundation Trust, UK; Eating Disorder Outpatient Service, South London and Maudsley NHS Foundation Trust, UK; Eating Disorder Service, North East London NHS Foundation Trust, UK; Department of Psychological Medicine, Institute of Psychiatry, Psychology and Neuroscience, King's College London, UK; Eating Disorder Outpatient Service, South London and Maudsley NHS Foundation Trust, UK; and Maudsley Health Eating Disorder Service, Maudsley Health, United Arab Emirates; Vincent Square Eating Disorder Service, Central and North West London NHS Foundation Trust, UK; Eating Disorder Service, Leeds and York Partnership NHS Foundation Trust, UK; Eating Disorder Service, North East London NHS Foundation Trust, UK; and Division of Psychology and Language Sciences, University College London, UK; Department of Psychological Medicine, Institute of Psychiatry, Psychology and Neuroscience, King's College London, UK; and Eating Disorder Outpatient Service, South London and Maudsley NHS Foundation Trust, UK

**Keywords:** Eating disorders, early intervention, emerging adults, anorexia nervosa, bulimia nervosa

## Abstract

**Background:**

The First Episode Rapid Early Intervention for Eating Disorders (FREED) service model is associated with significant reductions in wait times and improved clinical outcomes for emerging adults with recent-onset eating disorders. An understanding of how FREED is implemented is a necessary precondition to enable an attribution of these findings to key components of the model, namely the wait-time targets and care package.

**Aims:**

This study evaluated fidelity to the FREED service model during the multicentre FREED-Up study.

**Method:**

Participants were 259 emerging adults (aged 16–25 years) with an eating disorder of <3 years duration, offered treatment through the FREED care pathway. Patient journey records documented patient care from screening to end of treatment. Adherence to wait-time targets (engagement call within 48 h, assessment within 2 weeks, treatment within 4 weeks) and care package, and differences in adherence across diagnosis and treatment group were examined.

**Results:**

There were significant increases (16–40%) in adherence to the wait-time targets following the introduction of FREED, irrespective of diagnosis. Receiving FREED under optimal conditions also increased adherence to the targets. Care package use differed by component and diagnosis. The most used care package activities were psychoeducation and dietary change. Attention to transitions was less well used.

**Conclusions:**

This study provides an indication of adherence levels to key components of the FREED model. These adherence rates can tentatively be considered as clinically meaningful thresholds. Results highlight aspects of the model and its implementation that warrant future examination.

Rapid access to early intervention services in psychiatry can result in better outcomes and higher patient satisfaction, compared with treatment-as-usual (TAU) approaches.^1^ One such service is First Episode Rapid Early Intervention for Eating Disorders (FREED), designed for emerging adults (aged 16–25 years) with recent-onset eating disorders.^2^ Eating disorders are associated with substantial physical and psychosocial morbidity,^3^ and over time can become less amenable to change.^4–6^ Emerging adulthood is a peak risk period for eating disorder onset, yet evidence suggests that help-seeking and treatment utilisation are particularly low within this group.^7–9^ FREED aims to deliver developmentally informed care for emerging adults that reduces service-related delays and barriers to treatment, to maximise the likelihood of recovery and minimise the impact on psychosocial trajectories.

## FREED service model

FREED operates as a service within a service, overseen by a FREED Champion (typically a psychologist or nurse) who coordinates and leads a mini-team of clinicians delivering FREED-adapted treatment. Procedurally, the model involves wait-time targets of 2 weeks for assessment and 4 weeks for treatment, an electronic patient tracker to monitor and manage patient throughput, and weekly FREED ‘huddles’ and clinical supervision. Referrals to the service receive an engagement call within 48 h of referral. This aims to engage patients by validating and praising help-seeking, emphasising the importance of early intervention, and alleviating concerns (e.g. practical concerns, confidentiality concerns and fears about change and not being unwell enough to access treatment). Finally, the content of evidence-based treatment and style of working are adapted to meet the illness stage and developmental needs of emerging adults with recent-onset eating disorders. Treatment is delivered in a person-centred, motivational and flexible style, with a focus on transitions, eating disorder-related brain changes, social media use and significant other involvement.^10^

## FREED implementation and evidence base

The implementation and evaluation of FREED has been guided by the RE-AIM (Reach, Effectiveness/Efficacy, Adoption, Implementation, Maintenance) framework.^10,11^ This framework highlights five key dimensions that facilitate or hinder the population-based impact of an intervention. These dimensions are (a) the reach to the target population; (b) the effectiveness/efficacy; (c) the adoption of the intervention by organisations or individuals that can deliver it; (d) the implementation fidelity, time and cost and (e) the maintenance of an intervention over time.^12^ An overview of the implementation of FREED to date, with reference to the RE-AIM framework, is provided by Allen et al.^10^ The effectiveness of FREED has been demonstrated in a single-site pilot study (*N* = 142) and a larger multi-site study (FREED-Upscaled (FREED-Up) study; *N* = 502). Specifically, FREED increases treatment uptake, and reduces wait times and duration of untreated eating disorder (i.e. time between the onset of an eating disorder and the start of evidence-based treatment). It also improves eating disorder symptoms and reduces the need for costly in-patient/day treatment, compared with TAU.^13–15^ The successful and ongoing scaling of FREED to eating disorder services across England and internationally, alongside active outreach with community stakeholders and FREED's online presence, all continue to build toward the reach and adoption of FREED.^10^

Once an effective intervention is adopted across a growing number of settings and organisations, it is important to ensure that it is delivered as intended, i.e. implementation fidelity.^11^ Fidelity can mediate treatment effects and explain why an intervention is more successful in one setting than another.^16^ Evaluations of fidelity also provide valuable information regarding the feasibility of an intervention and where additional training and support may be needed. To date, there has been limited evaluation of the implementation dimension for FREED. Here, we focus on evaluating one component of this dimension, namely, adherence to key aspects of the model during the multi-site FREED-Up study: the wait-time targets and the FREED care package. The wait-time targets for the engagement call (<48 h), assessment (<2 weeks) and treatment (<4 weeks) are advisory and aspirational rather than obligatory. Although wait-time targets can reduce the wait for care,^17,18^ they can have unintended consequences, such as tunnel vision (i.e. a focus on the target to such an extent that other important features of healthcare are neglected).^19^ Target implementation requires careful consideration and ongoing evaluation to ensure that they are challenging and clinically meaningful but also achievable.^20^ The FREED care package tailors treatment to the needs of emerging adults with recent-onset eating disorders. In evaluations of FREED to date, it is unclear to what extent the care package adaptations were actually used and contributed toward the positive outcomes in the FREED-Up study. The care package adaptations measured in the FREED-Up study are outlined in Table 1.

**Table 1:** FREED care package adaptations in the FREED-Up study

Adaptation	Description
Biological malleability rationale for early intervention	A focus on the malleability of brain changes associated with eating disorders, emphasising the need for early intervention to restore brain changes and enhance the likelihood of recovery.
Psychoeducation on the impact of eating disorders on brain, body and behaviour	Verbal and/or written psychoeducation materials on the impact of eating disorders on the brain, body and behaviour, initiated early at assessment and continued throughout treatment (e.g. the psychological effects of starvation, and the vicious cycle of dieting, bingeing and purging) – even more than in treatment as usual, with tailoring to developmental stage.
Dietary change	A focus on dietary change initiated early at assessment, with initial goal setting and meal planning, and during treatment, with nutritional information, meal planning, goal setting and, where possible, early dietetic involvement.
Family/significant other involvement	Active and ongoing encouragement for family or significant other involvement in care that is developmentally appropriate and collaboratively planned. Where possible, discussions around carer skills training and support should be provided.
Exploration of social media and health-related app use	An exploration of social media and health-related app use as a potential maintaining factor for the eating disorder at assessment and treatment. A ‘Social Media and Apps – Friends or Foes?’ booklet can be given to patients.
Exploration of transitions	Special attention is given to the experience and management of transitions in care and life. Structured university preparation groups, covering topics such as social and sexual health, budgeting, time management, cooking and developing independence, can also be provided by teams.

FREED, First Episode Rapid Early Intervention for Eating Disorders; FREED-Up, First Episode Rapid Early Intervention for Eating Disorders - Upscaled.

The present study addressed three questions. First, how closely were the FREED wait-time targets for the engagement call, assessment and treatment adhered to, and did this vary across treatment group (FREED versus TAU) or diagnoses? Second, how frequently were the FREED care package adaptations used at assessment and during treatment, and did this use vary across diagnoses? Third, did the use of the FREED care package adaptations change throughout treatment?

## Method

### Study design and sample

This study is an analysis of patient journey record (PJR) data collected during the FREED-Up study. In brief, FREED-Up was a multi-site, quasi-experimental, pre–post study evaluating the impact of FREED compared with TAU on wait times, duration of untreated eating disorder and clinical outcomes (study findings are detailed elsewhere^10,13^). The study took place across four large specialist National Health Service (NHS) eating disorder out-patient services in England. Ethical approval was granted by the Camberwell St Giles Research Ethics Committee (16/LO/1882) and NHS Health Research Authority. The study was conducted in accordance with the ethical standards of relevant national and institutional committees on human experimentation and with the Helsinki Declaration of 1975, as revised in 2008.

FREED patients (*n* = 278) were aged 16–25 years, had a primary diagnosis of an eating disorder (according to DSM-5 criteria) and an eating disorder illness duration of <3 years. Diagnosis and illness duration were determined by a structured interview based upon the Eating Disorder Diagnostic Scale^21^ and the Eating Disorder Examination.^22^ Illness duration was operationalised as the time since the onset of a diagnosable eating disorder. Exclusion criteria were need for immediate in-patient admission, a comorbid physical or mental disorder that should be the primary focus of treatment, and a severe intellectual disability or insufficient English language ability to complete study procedures. Written informed consent was obtained from all participants. The TAU comparison group (*n* = 224) were patients aged 16–25 years with an eating disorder illness duration of <3 years who were referred to the eating disorder services during the 1.5- to 2-year period before the implementation of FREED. Electronic patient records were screened to identify TAU patients that were of comparable age and illness duration to FREED patients. The present study largely focused on data from FREED patients with PJRs. However, wait-time data for TAU were included for comparison purposes.

### Outcomes

#### Sample characteristics

Sociodemographic and Eating Disorder Examination Questionnaire (EDE-Q) data were collected at baseline. The EDE-Q^23^ is a 36-item questionnaire measuring attitudinal and behavioural aspects of eating disorders in the past 28 days. Only the EDE-Q global score is reported here. The global score consists of 22-items covering the domains of dietary restraint, eating concerns, and concerns about weight and shape. Each item is rated on a seven-point scale for severity or frequency, with higher scores indicating greater eating disorder psychopathology.

#### Wait times

Wait times for the engagement call, assessment and treatment were defined as the time from when the referral was received by the service to when the patient received the engagement call, attended the assessment or attended the first treatment session. Estimates of the average wait times are reported elsewhere.^13^ Here, count data of the number of patients seen within the FREED timeframes were used: ≤2 working days for the engagement call (i.e. calculation excluded weekends), ≤14 days for assessment and ≤28 days for treatment. Additionally, count data for the number of patients whose engagement call was initially attempted within 2 days (irrespective of whether it was successful or not), and the number of patients initially offered an assessment in ≤14 days or treatment in ≤28 days (regardless of whether the patient accepted the appointment or not), were included. Understanding waits that go beyond the initial timelines could prove informative for understanding any delays and for the development of the FREED model in the future. For this reason, count data for the number of patients seen within extended versions of the wait-time targets were also included, in the form of participants seen within 4 weeks (28 days) for assessment and 8 weeks (56 days) for treatment.

#### PJRs

Data from PJRs, developed for the study and completed by clinicians, were used here. PJRs documented the care received by FREED patients from referral up to 1 year. The form records service process data such as date of referral, screening call, and assessment and treatment sessions. It also details (a) the type of evidence-based out-patient psychological intervention provided (i.e. cognitive–behavioural therapy for eating disorders, the Maudsley Model of Anorexia Nervosa Treatment for Adults (MANTRA), guided self-help), for how many sessions; and (b) whether and when FREED-related care package adaptations were provided at assessment or treatment (see Table 1). The form also records any other additional out-patient appointments (e.g. dieticians sessions, medical reviews). Only the frequency of these additional appointments was reported, and not their content, as these were assumed to have a specific purpose (e.g. meal planning in dietician sessions or risk assessment in medical reviews).

### Analysis

Statistical analyses were conducted with R programming software version 4.0.5 for MacOS.^24^ The frequency (percentage) of adherence to the wait-time targets and the overall use of care package components at assessment and treatment are reported. Changes in the use of care package adaptations over time were also evaluated by calculating the frequency of use at different stages of treatment. For this, treatment was categorised into five stages: stage 1, sessions 1–5; stage 2, sessions 6–10; stage 3, sessions 11–15; stage 4, sessions 16–25 and stage 5, session 26 to end of treatment. For wait-time targets, the key focus was on adherence to the set FREED timelines (i.e. 48 h for engagement call, 2 weeks for assessment, 4 weeks for treatment) and adherence to an extended version of this timeline (i.e. 4 weeks for assessment and 8 weeks for treatment).

Chi-squared or Fisher's exact tests were used as appropriate, to evaluate whether there were any significant variations in wait-time adherence and care package use across diagnostic groups and treatment group. Moreover, we conducted an analysis of the differences in wait-time adherence between patients who did and did not receive FREED under optimal conditions. Patients with optimal conditions had minimal external delays (no gatekeeping or patient-related delays, such as patients taking a holiday before commencing treatment), no prior treatment and/or no transitions from another service. *Post hoc* analyses of the adjusted standardised residuals were used to determine which categories had substantially larger or smaller frequencies than expected, in the context of a significant omnibus test. In accordance with statistical conventions, a standardised residual of ±1.96 or more was considered as significant.^25^ For continuous variables, a robust alternative to the *t*-test, the Yuen–Welch test *T_y_*, based upon 10% trimmed means and Winsorized variances alongside percentile-t bootstrapping (2000 bootstrap samples), was used.^26^

## Results

### Sample characteristics

PJRs were available for 259 out of 278 (93%) FREED patients in the FREED-Up study. The demographics and clinical characteristics of the patients with PJRs are presented in Table 2. Patients with PJRs did not significantly differ from those without PJRs in age, gender, ethnicity, baseline EDE-Q global score and wait from referral to assessment or treatment (*P*-values varied from 0.16 to 1). Only data from patients with PJRs were included in the subsequent analyses.

**Table 2:** Baseline characteristics of FREED patients, with patient journey records

	Anorexia nervosa, *n* = 109	Bulimia nervosa/binge eating disorder, *n* = 69	Other specified feeding or eating disorder, *n* = 81	All, *N* = 259
Age, years, mean (s.d.)	19.88 (2.09)	20.62 (2.31)	20.22 (2.63)	20.19 (2.34)
Gender (female:male)	105:4	66:3	70:11	241:18
Ethnicity, *n* (%)				
White	75 (69)	36 (52)	59 (73)	170 (66)
Asian	10 (9)	8 (12)	7 (9)	25 (10)
Black	3 (3)	4 (6)	3 (4)	10 (4)
Mixed	6 (6)	10 (15)	3 (4)	19 (7)
Other/unknown	15 (14)	11 (16)	9 (11)	35 (14)
EDE-Q, mean (s.d.)	3.69 (1.43)	4.38 (0.90)	4.28 (1.07)	4.06 (1.23)

FREED, First Episode Rapid Early Intervention for Eating Disorders; EDE-Q, Eating Disorder Examination Questionnaire.

### Wait-time target adherence

Adherence to FREED wait-time targets is shown in Table 3, along with the percentage of FREED patients who received an assessment and treatment according to extended (4 and 8 weeks, respectively) wait-time targets. The engagement call was initially attempted within 48 h for 89% of patients, with approximately 50% actually receiving the call within this time, irrespective of diagnosis (attempted: *χ*^2^(2) = 2.18, *P* = 0.34; received: *χ*^2^(2) = 0.54, *P* = 0.76) or whether they received FREED under optimal conditions (attempted: *χ*^2^(1) = 0.01, *P* = 0.90; received: *χ*^2^(1) = 1.01, *P* = 0.31).

**Table 3:** Adherence to service wait-time targets for all patients and patients with optimal conditions

	FREED, all patients				FREED, patients with optimal conditions			
	Anorexia nervosa	Bulimia nervosa/binge eating disorder	Other specified feeding or eating disorder	All	Anorexia nervosa	Bulimia nervosa/binge eating disorder	Other specified feeding or eating disorder	All
Engagement call, *n* (%)								
Attempted ≤48 h	93/101 (92)	53/59 (90)	63/74 (85)	209/234 (89)	50/54 (93)	42/47 (89)	36/42 (86)	128/143 (90)
Received ≤48 h	53/100 (53)	32/66 (49)	36/75 (48)	121/241 (50)	26/55 (47)	24/50 (48)	20/42 (48)	70/147 (48)
Assessment, *n* (%)								
Offered ≤2 weeks	54/104 (52)	36/63 (57)	36/78 (46)	126/245 (51)	35/55 (64)	31/48 (65)	20/42 (48)	86/145 (59)
Received ≤2 weeks	50/109 (46)	30/69 (44)	30/81 (37)	110/259 (43)	30/55 (55)	28/55 (55)	17/43 (40)	75/149 (50)
Received ≤4 weeks^a^	78/109 (72)	49/69 (71)	61/81 (75)	188/259 (73)	45/55 (82)	43/51 (84)	38/43 (88)	126/149 (85)
Treatment, *n* (%)								
Offered ≤4 weeks	40/100 (40)	20/63 (32)	18/76 (24)	78/239 (33)	23/52 (44)	17/46 (37)	10/42 (24)	50/140 (36)
Received ≤4 weeks	28/108 (26)	15/69 (22)	17/79 (22)	60/256 (23)	17/54 (32)	14/51 (28)	10/41 (24)	41/146 (28)
Received ≤8 weeks^a^	64/108 (59)	41/69 (59)	42/79 (53)	147/256 (57)	40/54 (74)	35/51 (69)	26/41 (63)	101/146 (69)

All comparisons were made across diagnosis for all FREED patients and patients with optimal conditions. FREED, First Episode Rapid Early Intervention for Eating Disorders.

a Extended wait-time targets.

Overall, 51% of FREED patients were offered an assessment within 2 weeks and 43% of FREED patients actually received their assessment within 2 weeks. This was substantially higher than the TAU patients (*χ*^2^(1) = 30.06, *P* < 0.001). Only 19% of TAU patients were seen for assessment within 2 weeks. Diagnostic group did not affect whether FREED patients were offered or seen within 2 weeks for assessment (offered: *χ*^2^(2) = 1.70, *P* = 0.43; received: *χ*^2^(2) = 1.52, *P* = 0.47). The number of patients waiting <2 weeks increased significantly for offered (*χ*^2^(1) = 8.83, *P* < 0.01) and attended (*χ*^2^(1) = 8.88, *P* < 0.01) assessments if patients were seen under optimal conditions.

A total of 33% of FREED patients were offered treatment within 4 weeks and 22% started treatment within 4 weeks. Again, this was substantially higher than the TAU group, with only 3% of this group starting treatment within 4 weeks (*χ*^2^(1) = 30.10, *P* < 0.001). Slightly more FREED patients with anorexia nervosa were offered treatment within 4 weeks, compared with bulimia nervosa/binge eating disorder and other specified feeding or eating disorder; however, this difference did not reach statistical significance (offered: *χ*^2^(2) = 5.26, *P* = 0.07). Diagnostic group did not affect the number of FREED patients attending treatment within 4 weeks (received: *χ*^2^(2) = 0.65, *P* = 0.72). Receiving FREED under optimal conditions significantly increased the likelihood of being seen within 4 weeks (received: *χ*^2^(1) = 4.08, *P* = 0.04), but did not significantly affect the number of patients offered treatment within this timeframe (offered: *χ*^2^(1) = 1.46, *P* = 0.29).

Extending the wait-time targets for received assessment and treatment to 4 and 8 weeks resulted in a considerable increase in adherence rates, to 73% and 58%, respectively. The increase in adherence was even more striking for offered assessment and treatment appointments (80% and 67%), or if patients with external delays were excluded (85% and 69%).

### Care package adherence

#### Assessment

Assessment data were available for 241 out of 259 (93%) FREED patients with PJRs. As Table 4 shows, most domains of the FREED care package were well used at assessment, with the exception of attention to transitions. Highly used adaptations included a verbal discussion about the impact of eating disorders on brain, body and behaviour, followed by a verbal discussion of social media use, any discussion of or actual involvement of family/significant others, and the biologically malleability rationale for early intervention. The accompanying online or print resources were less frequently used. Any focus on dietary change occurred in approximately half of all assessments. In accordance with the FREED model, the most widely used components of dietary change at assessment were early nutritional goal setting and meal planning. In relation to significant other involvement, a discussion about involvement was the most frequently reported adaptation, followed by a significant other actually attending the assessment. The significant other most frequently attending the assessment were mothers (57%), followed by romantic partners (11%), parents (9%), siblings (7%), friends (7%) and fathers (5%).

**Table 4:** Percentage of patients receiving care package adaptations at assessment and treatment

Adaptations	Anorexia nervosa		Bulimia nervosa/binge eating disorder		Other specified feeding or eating disorder		All	
	Assessment, *n* = 102	Treatment, *n* = 106	Assessment, *n* = 64	Treatment, *n* = 68	Assessment, *n* = 75	Treatment, *n* = 77	Assessment, *n* = 241	Treatment, *n* = 251
Biological malleability rationale for early intervention	80%	49%**	67%	38%	83%	25%**	78%	39%
Psychoeducation on the impact of eating disorders								
Verbal discussion	88%	85%**	88%	96%	87%	96%	88%	91%
Leaflet or online resources given/reviewed	35%	28%	30%	35%	36%	35%	34%	32%
Dietary change								
Any focus on dietary change	58%	98%	41%*	100%	59%	99%	53%	99%
Nutrition booklet given/reviewed	25%**	40%	13%	38%	11%	52%	17%	43%
Meal plan given/reviewed	21%**	82%	6%	85%	11%	74%	14%	81%
Other nutrition information given/reviewed	11%	53%	6%	52%	8%	46%	9%	50%
Nutritional goal set/reviewed	23%	81%	9%	82%	23%	91%	19%	85%
Dietician appointment discussed/made	4%	45%**	2%	25%*	3%	29%	3%	35%
Dietician or dietetic group attended	Not applicable	63%***	Not applicable	25%**	Not applicable	26%***	Not applicable	41%
Family/carer/significant other involvement								
Any focus on significant other involvement	85%	90%***	72%	74%	78%	70%**	80%	79%
Discussed significant other involvement	63%	82%***	48%	63%	55%	56%**	56%	69%
Significant other attended assessment or treatment	40%**	55%***	13%***	25%*	33%	21%***	31%	36%
Discussed carer skills training	27%	39%***	16%	16%*	16%	14%**	20%	25%
Discussed carer support	33%	41%***	17%	21%	24%	12%***	26%	26%
Discussed family therapy	9%	23%	13%	18%	8%	13%	10%	18%
Family session attended	Not applicable	16%	Not applicable	9%	Not applicable	7%	Not applicable	11%
Discussed multi-family therapy	0%	1%	2%	3%	0%	1%	0.4%	2%
Exploration of social media and health-related app use								
Verbal discussion	78%*	53%	86%	62%	92%*	57%	84%	57%
Social media booklet/resources given/reviewed	35%	27%	31%	22%	28%	36%	32%	29%
Exploration of transitions								
Verbal discussion	27%	49%	34%	35%	36%	47%	32%	45%
University preparation group recommended	3%	22%	3%	10%	3%	12%	3%	16%
University preparation group attended	Not applicable	6%	Not applicable	0%	Not applicable	4%	Not applicable	4%

*P < 0.05, **P < 0.01, ***P < 0.001.

There were significant differences in assessment adaptation use across diagnoses, as indicated in Table 4. Specifically, any focus on dietary change was less likely in bulimia nervosa/binge eating disorder relative to anorexia nervosa and other specified feeding or eating disorder (*χ*^2^(2) = 5.84, *P* < 0.05). Compared with patients with bulimia nervosa/binge eating disorder or other specified feeding or eating disorder, patients with anorexia nervosa were substantially more likely to receive the nutritional booklet (*χ*^2^(2) = 7.12, *P* < 0.05) and meal planning (*χ*^2^(2) = 7.68, *P* < 0.05) at assessment. Patients with anorexia nervosa were also more likely to have a significant other attend the assessment than patients with bulimia nervosa/binge eating disorder (*χ*^2^(2) = 14.53, *P* < 0.001). Finally, social media use was more frequently explored in other specified feeding or eating disorder, and less frequently explored in anorexia nervosa (*χ*^2^(2) = 7.07, *P* < 0.05).

#### Treatment

Treatment data were available for 251 out of 259 (97%) FREED patients with PJRs. The average number of treatment sessions was 18.09 (s.d. 11.70, range 0–57), with anorexia nervosa receiving more sessions (mean 22.83, s.d. 12.74), compared with bulimia nervosa/binge eating disorder (mean 14.10, s.d. 8.34) and other specified feeding or eating disorder (mean 15.03, s.d. 10.44). Patients with anorexia nervosa received cognitive–behavioural therapy for eating disorders (49%), MANTRA (48%), cognitive analytic therapy (6%) or family-based therapy (1%). Patients with bulimia nervosa/binge eating disorder received cognitive–behavioural therapy for eating disorders (83%), guided self-help (9%) or cognitive analytic therapy (3%). Patients with other specified feeding or eating disorder received cognitive–behavioural therapy for eating disorders (90%), MANTRA (6%), family-based therapy (3%) or cognitive analytic therapy (2%).

**Fig. 1:**
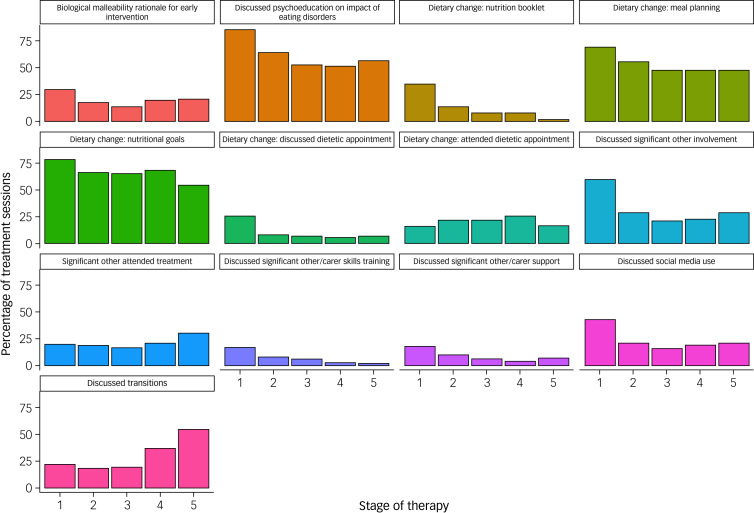
The frequency (percentage of sessions) of use of FREED treatment adaptations across stages of therapy. Stage 1: sessions 1–5; stage 2: sessions 6–10; stage 3: sessions 11–15; stage 4: sessions 16–25; stage 5: session 26 to end of treatment. All *x*-axes represent stages 1–5 of therapy. FREED, First Episode Rapid Early Intervention for Eating Disorders.

Table 4 shows the overall use of care package adaptations during treatment, and Fig. 1 depicts the change in adaptation use over time. Similar to assessment, psychoeducational discussions on the impact of eating disorders on brain, body and behaviour remained high throughout treatment. In contrast, the biological malleability rationale was less frequently used during treatment, relative to assessment. Social media and health-related app use was also less frequently explored in treatment relative to assessment, with most discussions occurring within the first five sessions of treatment (43% at stage 1 *v.* 21% at stage 5). The use of accompanying online and print resources remained low during treatment, with the exception of the nutrition booklet, which was used more during treatment relative to assessment. The most highly used domain of the care package during treatment was any focus on dietary change. Among the dietary change activities, nutritional goal setting and meal planning were the most frequently used. Approximately 40% of patients saw a dietician individually or in a group setting at some point during treatment.

Overall, any type of significant other involvement remained high during treatment. Discussions about significant other involvement and actual attendance were the most frequently used carer-related activities. Carer support and skills training were less frequently used. Most carer-related activities occurred within the first five sessions of treatment, with the exception of attendance, which peaked at stage 5. There were limited discussions of family and multi-family therapy, and family sessions taking place. Similar to assessment, mothers tended to be the person who most frequently attended the treatment sessions (47%), followed by parents, families or fathers (37%), and others (16%). Attention to transitions increased during treatment relative to assessment; however, discussions of or use of the university preparation groups remained low. Unlike most adaptations, use of attention to transitions steadily increased over the course of treatment (22% at stage 1 *v.* 55% at stage 5).

As highlighted by the asterisks in Table 4, patients with anorexia nervosa were significantly more likely to have discussions around dietetic involvement (*χ*^2^(2) = 9.34, *P* < 0.01), attendance to dietetic appointments or groups (*χ*^2^(2) = 35.86, *P* < 0.001), any type of significant other involvement (*χ*^2^(2) = 12.20, *P* < 0.01), discussions around significant other involvement (*χ*^2^(2) = 15.74, *P* < 0.001), significant other attendance at treatment (*χ*^2^(2) = 27.34, *P* < 0.001), and discussions around carer skills training (*χ*^2^(2) = 18.07, *P* < 0.001) and support (*χ*^2^(2) = 20.76, *P* < 0.001). Moreover, patients with anorexia nervosa were more likely to receive the biological malleability rationale for early intervention during treatment (*χ*^2^(2) = 11.19, *P* < 0.01). In contrast, patients with bulimia nervosa/binge eating disorder and other specified feeding or eating disorder were significantly more likely to receive psychoeducation on the impact of eating disorders than anorexia nervosa (*χ*^2^(2) = 9.20, *P* < 0.01), but use was high across all groups.

## Discussion

The process of translating new interventions into real-world clinical settings is complicated. The RE-AIM framework, a tool for enhancing the implementation and generalisability of interventions, was used to support the translation of FREED from a single-site research project to a wider initiative, with the aim of reaching as many young people as possible.^10^ The purpose of this study was to evaluate the implementation dimension of the RE-AIM framework in the multi-site FREED-Up study. Specifically, we evaluated adherence to two key components of the model during the study, the wait-time targets and the care package, and whether adherence varied over time or across diagnostic and treatment group.

### Wait-time targets

Most patients, irrespective of diagnosis, had their engagement call attempted within 48 h, with approximately half receiving the call within this timeframe. This suggests that although the 48 h target is a realistic goal for services, actually getting the patient on the telephone can be challenging. Patients frequently require multiple telephone calls, may not feel comfortable talking over the telephone, or may be ambivalent or refuse to engage with clinicians. Ambivalence can be particularly problematic in early-stage illness, where the negative physiological and psychosocial consequences of eating disorders may not be as apparent to the young person.^7^ To overcome these barriers, FREED advocates for a flexible and proactive approach when engaging patients via their preferred method of contact (e.g. email, text). Specifically, if initial engagement attempts were unsuccessful, clinicians tried different methods of contact, with a higher number of attempts over a longer period of time than traditionally used in services (i.e. did more ‘chasing’). Once contact was established, patients were also asked what method of contact they would prefer. This provides patients with a greater sense of autonomy in how they communicate with the service.

There was moderate adherence to the 2-week wait-time target for assessment, and low adherence to the 4-week wait-time target for treatment. However, the introduction of FREED led to large increases in the number of patients seen within these timeframes. Double the number of patients were seen within 2 weeks for assessment, and almost ten times as many patients were seen within 4 weeks for treatment. Substantial differences were also evident between offered and attended appointments for those with and without external delays, suggesting that external and patient-related factors require special attention when addressing delays to care. Patient-related delays could be addressed through evidence-based public awareness campaigns^9^ and the development of tools, apps and online resources to support emerging adults to seek help earlier. There was also a trend toward patients with anorexia nervosa being more likely to be offered treatment within 4 weeks.

This study provides an indication of the percentage of patients that teams can expect to see within the wait-time targets in real-world clinical settings: approximately 90% for attempted engagement calls in <48 h, approximately 60% for an assessment offered in <2 weeks, and approximately 30% for treatment offered in <4 weeks. This level of adherence was associated with significant reductions in wait times and duration of untreated eating disorder relative to TAU,^13^ suggesting that these adherence rates are clinically meaningful irrespective of whether the targets were achieved or not. However, barriers to adherence need to be addressed in the future implementation of FREED. Targets should be challenging, but also realistically achievable with the available skills and resources. Unattainable targets can motivate in the short term, but eventually lead to frustration and stress.^27,28^ Additional resources or an extension of the wait-time targets may therefore be warranted for some teams who are using FREED. Extending targets for assessment and treatment to 4 and 8 weeks, respectively, led to vast improvements in adherence rates, and may thus serve as achievable interim targets.

Our findings are timely, given recent commitments by NHS England to introduce access and wait-time standards for mental health services.^29^ Wait-time standards of treatment within 4 weeks from referral for routine cases and 1 week for urgent cases have already been introduced in child and adolescent eating disorder services (CAEDS).^30^ In the second quarter of 2020/21, 85% of referrals started urgent treatment within a week and 90% started routine treatment within 4 weeks. Approximately 65% were seen within these targets when they were first introduced in 2016.^31^ Considerable and continued investment in CAEDS (an additional £30 million funding a year in the first instance, and a further £11 million in 2019/20 and 2020/21), rigorous performance monitoring and a national programme of training and support were vital to enable such vast improvements in target adherences. Our study provides the first evaluation of adherence to wait-time targets in adult eating disorder services, but with very limited government investment to date.^32,33^ Of note, the CAEDS waiting time targets use initial assessment as the start of treatment, which is more lenient than our separate assessment and treatment targets. If we apply this more lenient criterion here, around 70% of our FREED-Up patients would have been seen within the target period.^13^ These findings must be seen against the wider backdrop of resource constraint within adult eating disorder services in the NHS, something that is only likely to be exacerbated by the ongoing COVID-19 pandemic.^34^

### Care package

Overall, the care package adaptations were well used during the FREED-Up study, increasing confidence in the extent to which this aspect of the model facilitates positive outcomes. The overarching domains were highly used at assessment or treatment, with the exception of attention to transitions, which was used in approximately half of all cases at either stage of care. This may be understandable, given that not all patients will experience transitions when in treatment, despite the relevance of transitions to the emerging adult developmental stage. Attention to transitions did, however, increase over the course of treatment, probably owing to the increased likelihood of transitions in later stages of treatment. Most other adaptations had a pattern of decreasing use over time, which is anticipated as once a topic is addressed it may not be necessary or appropriate to continue with it. Moreover, the therapeutic focus often becomes broader in the later stages of eating disorder treatment.^35,36^ However, attendance by significant others peaks in the last stage of treatment. This could be because of the type of patients (mainly anorexia nervosa) receiving over 25 sessions of treatment, or because it takes time to persuade young people to involve significant others.

Any focus on dietary change and psychoeducation were the most used adaptations in treatment. This is reassuring, given that nutritional rehabilitation is central to any evidence-based eating disorder treatment. However, dietary change activities were only moderately used at assessment, which is disappointing because early nutritional change is one of the primary principles of FREED. Limited use of dietary change activities at assessment could be because of patient-related ambivalence, clinician reservations and/or time constraints in the assessment session.

Some components of the care package had low-to-moderate use, specifically, accompanying print/online resources, discussions of family or multi-family therapy, carer skills training and support, and the university preparation groups. These components may be considered as more supplementary than other aspects of the care package, or may only have been discussed if the eating disorder service could provide that facility. Increasingly, there is a trend toward not just online, but also app-based or interactive online materials, and revising FREED care package components accordingly may be helpful.

The use of care package adaptations varied across the diagnostic groups. Patients with anorexia nervosa were more likely than other diagnoses to receive a focus on early dietary change at assessment and dietetic involvement during treatment, as well as significant other involvement, particularly significant other attendance, support and skills training. Compared with bulimia nervosa/binge eating disorder, patients with other specified feeding or eating disorder also received a higher focus on early dietary change, possibly because of anorexia nervosa-type presentations within this group. Anorexia nervosa is typically (but not always) a more outwardly visible illness, which may influence the perceived need for early nutritional change and signify to others that the individual is unwell and requires support. In contrast, the shame and secrecy associated with other eating disorders may inhibit their disclosure, and therefore require more effort to encourage significant other involvement. This imbalance in provision of nutritional advice and support, and significant other involvement, needs to be considered further in the future implementation of FREED.

### Limitations

There are several limitations to the current study that require consideration when interpreting the results. First, care package adaptation use was only assessed by clinician self-report. Although clinician-reported fidelity is efficient and non-intrusive, there are concerns regarding the accuracy of this method. Some studies find weak-to-moderate agreement between clinician and observer estimates.^37^ Further validation of this mode of fidelity monitoring for FREED should be the focus of future research. Second, this study did not evaluate the way in which care package adaptations were used, i.e. the style and quality of delivery. Merely mentioning social media versus having an in-depth discussion about it as a maintaining factor are likely to have profoundly different effects on patient outcomes, but would be noted down equally on the PJR. Limited information on the quality of delivery also prevented any meaningful evaluation of the impact of these adaptations on outcome. Third, the non-randomised design limits the causal conclusions that can be drawn regarding the impact of FREED on wait-times target adherence.^13^ Finally, the data were collected within the context of a research study. It is unclear to what extent these adherence rates will generalise to settings outside of the study, or when FREED becomes ‘business as usual’.

In conclusion, this study evaluated the implementation of FREED, with attention to waiting time and care package adherence. To the best of our knowledge, this is the first evaluation of adherence to wait-time targets in adult eating disorder services, providing a benchmark not only for FREED, but for what might be possible in NHS eating disorder services. Our findings suggest that adherence to the FREED wait-time targets can be an achievable goal, but require ongoing monitoring and refinement to ensure that the selected targets closely align with the baseline capacity of each team. This study also sheds light on how much and at what point FREED care package adaptations were used. There was moderate-to-high use of these adaptations that varied over the stages of treatment and between diagnoses. This supports the applicability of FREED, and suggests that care package adaptations are an important part of how FREED improves clinical outcomes. However, further validation of adherence, the quality of delivery and its impact on outcomes is needed. A better understanding of adherence to key components of the FREED model (and evidence-based treatments more generally) is essential for conclusions regarding what is integral to its effectiveness, and what aspects of the model may need to be adapted or refined.

## Data Availability

The data that support the findings of this study are available from the corresponding author, U.S., upon reasonable request.
